# Development of Culture Medium for the Isolation of *Flavobacterium* and *Chryseobacterium* from Rhizosphere Soil

**DOI:** 10.1264/jsme2.ME15144

**Published:** 2016-04-20

**Authors:** Tomoki Nishioka, Mohsen Mohamed Elsharkawy, Haruhisa Suga, Koji Kageyama, Mitsuro Hyakumachi, Masafumi Shimizu

**Affiliations:** 1 Graduate School of Applied Biological Sciences, Gifu University1–1 Yanagido, Gifu City 501–1193Japan; 2 Department of Agricultural Botany, Faculty of Agriculture, Kafrelsheikh UniversityKafr Elsheikh 33516Egypt; 3 Life Science Research Center, Gifu University1–1 Yanagido, Gifu City 501–1193Japan; 4 River Basin Research Center, Gifu University1–1 Yanagido, Gifu City 501–1193Japan

**Keywords:** *Flavobacterium*, *Chryseobacterium*, Isolation medium, Rhizosphere soil

## Abstract

An effective medium designated phosphate separately autoclaved Reasoner’s 2A supplemented with cycloheximide and tobramycin (PSR2A-C/T) has been developed for the isolation of *Flavobacterium* and *Chryseobacterium* strains from the plant rhizosphere. It consists of Reasoner’s 2A agar (R2A) prepared by autoclaving phosphate and agar separately and supplementing with 50 mg L^−1^ cycloheximide and 1 mg L^−1^ tobramycin. A comparison was made among the following nine media: PSR2A-C/T, PSR2A-C/T supplemented with NaCl, R2A agar, R2A agar supplemented with cycloheximide and tobramycin, 1/4-strength tryptic soy agar (TSA), 1/10-strength TSA, soil-extract agar, Schaedler anaerobe agar (SAA), and SAA supplemented with gramicidin, for the recovery of *Flavobacterium* and *Chryseobacterium* strains from the Welsh onion rhizosphere. *Flavobacterium* strains were only isolated on PSR2A-C/T, and the recovery rate of *Chryseobacterium* strains was higher from PSR2A-C/T than from the eight other media. In order to confirm the effectiveness of PSR2A-C/T, bacteria were isolated from onion rhizosphere soil with this medium. *Flavobacterium* and *Chryseobacterium* strains were successfully isolated from this sample at a similar rate to that from the Welsh onion rhizosphere.

The plant rhizosphere harbors a large bacterial community consisting of taxonomically and functionally diverse species that play pivotal roles in many processes, such as nitrogen fixation, mineralization, the decomposition of organic matter, and degradation of hazardous and xenobiotic chemicals. In addition, some rhizobacterial isolates affect the growth and ecological fitness of their host plants (2, 46). The biological control of soil-borne plant diseases, bioremediation, and biofertilizers has been attracting increasing attention, and a more detailed understanding of the biological functions of rhizobacteria and plant–rhizobacteria interactions is needed. Recent advances in culture-independent molecular ecological surveys have gradually been revealing bacterial communities in the plant rhizosphere. The phylum *Bacteroidetes* is one of the bacterial lineages that is abundant in the rhizospheres of various plants (7, 28, 31, 43, 56). For example, Gardner *et al.* (7) reported that *Bacteroidetes* bacteria accounted for more than 65% of the total bacterial population in the broccoli rhizosphere.

Among the members of *Bacteroidetes*, the family *Flavobacteriaceae*, particularly the genera *Flavobacterium* and *Chryseobacterium*, were found to be the most abundant rhizobacteria in the rhizospheres of a wide variety of plants, such as sweet potato (26), tomato (31), cucumber (43), and wheat (56).

The strains of *Flavobacterium* and *Chryseobacterium* isolated from soil are known to have beneficial properties for agricultural and industrial use. Plant growth-promoting (PGP) effects are considered to be one of the main agricultural uses of these strains. Several *Flavobacterium* strains have been reported to possess PGP capabilities, such as the solubilization of inorganic phosphate and production of indole-3-acetic acid (IAA) and 1-aminocyclopropane-1-carboxylate (ACC) deaminase (23, 38). Some *Chryseobacterium* strains have also been shown to synthesize IAA and ACC deaminase (25).

Another property of rhizobacteria that is of potential agricultural utility is plant protection against pathogens. Several strains of *Flavobacterium* produce antimicrobial compounds, such as 2,4-di-*tert*-butylphenol and hydrogen cyanide (HCN) (36, 38). Some *Chryseobacterium* strains have the ability to produce HCN and antifungal antibiotics and stimulate plant immune systems (17, 25, 32, 56). Strains of *Flavobacterium* and *Chryseobacterium* have been industrially used as bioremediation agents. As bioremediation/biodegradation agents, these strains have the ability to produce polysaccharide-degrading enzymes, such as chitinase (14), mannanase (33), and amylase (27, 49). Furthermore, *Chryseobacterium* strains have been shown to deamidate proteins, such as carboxybenzoxy (Cbz)-Gln-Gly, casein, and keratin, and produce proteases (8, 48, 55). Strains of *Flavobacterium* and *Chryseobacterium* are also considered to play important roles in the degradation of aromatic compounds, such as carbendazim (54), polycyclic aromatic hydrocarbons (21), and 2,4-dinitrotoluene in soil (50).

A significantly large number of species assigned to *Flavobacterium* and *Chryseobacterium* have more recently been isolated from the rhizospheres of Panax notoginseng (“*F. notoginsengisoli*”) (57), switchgrass (“*F. nitrogenifigens*”) (13), bell pepper (*Flavobacterium* sp. strain F52) (18), eggplant (*C. solani*) (6), cempedak (*C. artocarpi*) (45), maize (*C. zeae*) (12), and peanut (*C. arachidis* and *C. geocarposphaerae*) (12), implying that rhizosphere environments potentially harbor novel *Flavobacterium* and *Chryseobacterium* strains that may be useful for agricultural and industrial purposes. Our preliminary microbial community analysis using next-generation sequencing technology revealed that the rhizospheres of *Allium* plants contained a large number of *Flavobacterium* and *Chryseobacterium* strains (2.4–10.9% and 0.4–2.4% of total bacterial populations, respectively) (Nishioka, T., *et al.* 2014. Abstracts for Phytopathological Society of Japan Annual Meeting. p. 103, Hokkaido, in Japanese). Although many *Flavobacterium* and *Chryseobacterium* strains have been isolated by commonly used media, such as Reasoner’s 2A (R2A) agar and tryptic soy agar (TSA) (11, 16, 51, 52, 53), the development of selective culture media is necessary for the efficient retrieval of *Flavobacterium* and *Chryseobacterium* strains as microbial resources. Therefore, the objective of the present study was to optimize medium compositions for the isolation of *Flavobacterium* and *Chryseobacterium* strains from rhizosphere soil.

## Materials and methods

### Rhizosphere soil samples

Rhizosphere soils of the Welsh onion (*Allium fistulosum* L.) cv. Kujo-hoso and onion (*A. cepa* L.) cv. Sharm were used as the source materials for the isolation of bacteria. The Welsh onion and onion were sown in plug trays containing commercial potting medium (Negi-baido: Takii, Kyoto, Japan) and grown for 90 and 40 d, respectively. Seedlings of both plants were transplanted into plastic pots (15 cm in diameter, 8-cm deep) filled with soil collected from a field on the experimental farm of the university and were grown for 70 d in a greenhouse at 20–25°C under natural light with regular watering and fertilizing. Three plants were uprooted and loosely adhering soil was removed from the roots by gentle shaking for the collection of rhizosphere soil. Three grams of the pooled roots of each plant (1 g of the root of each plant body) was then suspended in sterile distilled water (SDW) and shaken for 15 min on a rotary shaker at 150 rpm. Serial soil dilutions of the samples were prepared in SDW and used for the surface plating of agar plates as described below.

### Isolation trial 1: Comparisons of basal media

In this trial, six types of agar media were used: R2A agar (34), 1/4-strength TSA (Difco Laboratories, Detroit, MI, USA), 1/10-strength TSA, soil-extract agar (SEA) (22), Schaedler anaerobe agar (SAA) (Oxoid, Basingstoke, UK), and SAA supplemented with gramicidin (17.5 μg mL^−1^) (SAA-Gr) (37). R2A agar, 1/4-strength TSA, 1/10-strength TSA, and SEA are commonly used media for the isolation of a wide range of bacterial species including *Bacteroidetes* and SAA-Gr agar has been used to isolate *Bacteroidetes* bacteria from the gastrointestinal tract of mice.

A 100-μL aliquot of each serial dilution of Welsh onion rhizosphere soil was spread onto the surface of each medium in triplicate plates (90 mm in diameter). R2A agar and SEA plates were incubated at 25°C in the dark for 14 d. Plates of 1/4-strength and1/10-strength TSA were incubated at two different temperatures. One set of each medium plate was incubated at 25°C in the dark for 14 d and the other set was incubated at 30°C in the dark for 3 d. SAA and SAA-Gr plates were incubated at 35°C in the dark for 3 d. Well-isolated, discrete colonies showing a yellow/orange color on plates that yielded 10–100 colonies were selected and streaked using quadrant streaking on R2A agar plates (60 mm in diameter) because *Flavobacterium* and *Chryseobacterium* are known to produce bright yellow non-diffusible and non-fluorescent flexirubin pigments (1, 9, 39). After streaking, the plates were incubated at 25°C for 2 d. Single colonies were transferred to newly prepared R2A plates and incubated at 25°C for 3 d. Bacterial cells on the plates were mixed thoroughly with 1 mL of skim milk medium (100 g L^−1^ skim milk and 16.5 g L^−1^ L-glutamic acid monosodium salt), transferred into 1.5-mL Eppendorf tubes, and stored at −80°C until used.

### Isolation trial 2: The effects of antibiotics

As described below, it was only possible to isolate *Bacteroidetes* bacteria with a yellow/orange color from R2A medium in isolation trial 1. Accordingly, R2A medium was selected as the basal isolation medium in subsequent isolation trials. Although colonies of *Bacteroidetes* bacteria appeared on R2A, this medium allowed the growth of contaminants such as fungi and orange pigment-producing sphingomonads belonging to the class *Alphaproteobacteria*. Accordingly, in this trial, R2A agar was supplemented with the fungicide cycloheximide (50 μg mL^−1^) and bacteriocide tobramycin (1 μg mL^−1^) (R2A-C/T). Diluted suspensions of Welsh onion rhizosphere soil were spread onto the surface of each medium in triplicate plates (90 mm in diameter) and incubated at 25°C for 14 d. After being incubated, bacteria with a yellow-orange color were isolated and stored as described above.

### Isolation trial 3: The effects of separately autoclaved phosphate

*Flavobacterium* are sensitive to inhibitory compounds in media in which phosphate compounds are autoclaved together with agar (41). Accordingly, in this experiment, the ingredients of R2A medium were placed into two groups. Solution A was prepared using yeast extract (1.0 g L^−1^), tryptone (0.5 g L^−1^), peptone (1.5 g L^−1^), starch (1.0 g L^−1^), glucose (1.0 g L^−1^), and agar (30 g L^−1^). Solution B consisted of K_2_HPO_4_ (0.6 g L^−1^), MgSO_4_ (48 mg L^−1^), and sodium pyruvate (0.6 g L^−1^). Solutions A and B were autoclaved separately and then mixed at the same quantity in order to prepare the basal medium, designated phosphate separately autoclaved R2A (PSR2A) medium. Two types of R2A agar were prepared: R2A-C/T and PSR2A supplemented with cycloheximide (50 μg mL^−1^) and tobramycin (1 μg mL^−1^) (PSR2A-C/T). Diluted suspensions of Welsh onion rhizosphere soil were spread onto the surface of each medium in triplicate plates (90 mm in diameter) and incubated at 25°C for 14 d. After being incubated, bacteria with a yellow-orange color were isolated and stored as described above.

### Isolation trial 4: The effects of sodium chloride

Two types of PSR2A were prepared: PSR2A-C/T and PSR2A supplemented with cycloheximide (50 μg mL^−1^), tobramycin (1 μg mL^−1^), and 1% NaCl (PSR2A-C/T/N). Diluted suspensions of Welsh onion and onion rhizosphere soil were spread onto the surface of each medium in triplicate plates and incubated at 25°C for 14 d. After being incubated, bacteria with a yellow/orange color were isolated and stored as described above.

### 16S rRNA gene sequence analysis

The genomic DNA of each isolate was prepared using PrepMan Ultra sample preparation reagent (Applied Biosystems, CA, USA) according to the manufacturer’s instructions. The 16S rRNA gene of each isolate was amplified with 27f and 1492r primers (20) and TaKaRa Ex Taq (Takara Bio, Otsu, Shiga, Japan). The amplification conditions were 94°C for 1 min (initial denaturation), followed by 25 cycles at 94°C for 1 min, at 55°C for 1 min, and at 72°C for 2 min, with a final extension step at 72°C for 8 min. The amplification products were purified using the GenElute PCR Clean-Up Kit (Sigma, MO, USA). Cycle sequencing was performed with the Big Dye Terminator v3.1 Cycle Sequencing Kit (Applied Biosystems, CA, USA), 27f or 517r primers (30), and an ABI PRISM 3100 Genetic Analyzer (Applied Biosystems). The cycle sequencing conditions used were: at 96°C for 1 min (an initial denaturation), followed by 25 cycles at 96°C for 10 s, at 50°C for 5 s, and at 60°C for 4 min. Partial 16S rRNA gene sequences were compared using a BLAST search (http://ddbj.nig.ac.jp/blast/blastn) with the reference sequences in the DNA Data Bank of Japan (DDBJ) database. Information on the closest relatives of the 16S rRNA gene sequences of each strain was provided as supplemental Table 1. The partial 16S rRNA gene sequences (corresponding to bases 1 to 629 of *Escherichia coli* NBRC102203^T^ of the 16S rRNA gene) obtained from 14 strains related to *Flavobacterium* and 19 strains related to *Chryseobacterium*, isolated from Welsh onion and onion rhizosphere soil on PSR2A-C/T in isolation trials 3 and 4, were aligned with reference sequences using CLUSTALW (42). Neighbor-joining trees were constructed with MEGA version 6.06 (40), and 1,000 bootstrap replicates were used to generate a consensus tree.

### Nucleotide sequence accession numbers

The 16S rRNA gene sequences of 14 strains related to *Flavobacterium* and 19 strains related to *Chryseobacterium*, isolated from Welsh onion rhizosphere soil in isolation trials 3 and 4 and onion rhizosphere soil in isolation trial 4, on PSR2A-C/T were deposited in DDBJ with the following accession numbers: LC096252–LC096256, LC034259–LC034263, and LC034264–LC034267 (for *Flavobacterium* strains from Welsh onion rhizosphere soil in isolation trials 3 and 4, and onion rhizosphere soil in isolation trial 4, respectively), LC096244–LC096251, LC034273–LC034278, and LC034268–LC034272 (for *Chryseobacterium* strains from Welsh onion rhizosphere soil in isolation trials 3 and 4, and onion rhizosphere soil in isolation trial 4, respectively).

## Results and discussion

### Isolation trial 1: Comparisons of basal media

Six types of agar media were used to isolate bacteria from Welsh onion rhizosphere soil. The total bacterial colony counts in the triplicate plates of each medium are summarized in Table 1. Only yellow or orange-colored colonies were selected for further characterization by the 16S rRNA gene sequence analysis. The proportions of bacterial colonies with a yellow/orange color were markedly higher on R2A agar (39.1%) and 1/4-strength TSA (67.3%) than on the other media (0–1.8%). Accordingly, we identified 71 yellow/orange-colored isolates collected from R2A agar and 1/4-strength TSA plates based on partial 16S rRNA gene sequences (Table 2). None of the isolates obtained from 1/4-strength TSA medium were assigned to the phylum *Bacteroidetes*. In contrast, three genera of *Bacteroidetes*, including *Chryseobacterium*, were found on R2A agar medium. Based on the results from isolation trial 1, R2A agar was the most suitable basal medium for the isolation of *Bacteroidetes* strains.

### Isolation trial 2: The effects of antibiotics

Although R2A agar medium yielded the maximum number of *Bacteroidetes*-related strains (11% of yellow/orange-colored bacteria) among the six types of media tested, a significantly large number of strains associated with *Proteobacteria* (71%) and *Actinobacteria* (18%) were also retrieved. Furthermore, R2A agar medium allowed fungal contaminants (data not shown). These results led us to use the antibiotics tobramycin and cycloheximide in order to prevent the growth of Gram-negative bacteria and fungi, respectively. Tobramycin is a strong inhibitor of Gram-negative bacteria, including most *Sphingomonas* strains, but not most strains of *Flavobacterium* and *Chryseobacterium* (3–5, 15, 24, 44). Cycloheximide is known to be a strong inhibitor of a wide range of fungi and yeasts (35).

Fourteen yellow/orange-colored colonies appeared on the triplicate plates of R2A agar and R2A-C/T media (Table 2). In accordance with the results of isolation trial 1, six (43%) out of the 14 yellow/orange-colored isolates obtained from R2A agar were assigned to the genus *Sphingomonas*. In contrast, no *Sphingomonas* strains were found in R2A-C/T medium, and all of the yellow/orange-colored isolates were assigned to the genus *Niabella* of *Bacteroidetes*. *Niabella* strains are also known to produce flexirubin-type pigments (1, 47); therefore, difficulties are associated with distinguishing *Niabella* from *Flavobacterium* and *Chryseobacterium* based on colony color. Nevertheless, fungal contamination was prevented by using R2A-C/T plates (data not shown), suggesting that the addition of antibiotics is a convenient way by which to prevent the proliferation of rhizosphere-associated *Proteobacteria* and fungi.

One possible way to improve the recovery rate of target microorganisms on selective medium is to reduce environmental stress factors in the medium. Tanaka *et al.* (41) previously reported that certain inhibitory substances, such as hydrogen peroxide, were generated in media when phosphate compounds were autoclaved together with agar, and also that *Flavobacterium* may be highly sensitive to these inhibitory substances. Accordingly, R2A agar medium, prepared by autoclaving phosphate compounds and agar separately (designated PSR2A), was used as the basal medium in isolation trial 3.

### Isolation trial 3: The effects of separately autoclaved phosphate

The colony counts of yellow/orange-colored bacteria were 17 and 19 on triplicate plates of R2A-C/T agar and PSR2A-C/T media, respectively (Table 2). Accordingly, no *Sphingomonas* strains were found on R2A-C/T medium, and all yellow/orange-colored isolates were assigned to *Bacteroidetes*. Although no isolates assigned to the genus *Flavobacterium* were recovered from R2A-C/T plates, 5 (26%) out of the 19 isolates obtained from PSR2A-C/T plates were affiliated with the genus *Flavobacterium*. In addition, the proportion of isolates assigned to the genus *Chryseobacterium* was higher in PSR2A-C/T plates (42%) than in R2A-C/T plates (24%).

Strains of *Flavobacterium* and *Chryseobacterium* are often isolated from seawater (10, 24, 29) whereas, to the best of our knowledge, there have been no reports of marine *Niabella* strains. Accordingly, we hypothesized that *Flavobacterium* and *Chryseobacterium* have a higher salt tolerance than *Niabella* strains, and, thus, NaCl may be used as an inhibitor of *Niabella* strains.

### Isolation trial 4: The effects of sodium chloride

In this trial, two types of PSR2A media (PSR2A-C/T and PSR2A-C/T/N) were used for bacterial isolation. Serial dilutions of the rhizosphere soil of the Welsh onion were spread onto the surfaces of these media. The colony counts of the yellow/orange-colored bacteria were 20 and 15 on triplicate plates of PSR2A-C/T and PSR2A-C/T/N media, respectively (Table 2). Among the 20 isolates obtained from PSR2A-C/T plates, 16 (80%) were affiliated with the phylum *Bacteroidetes*. In contrast, nine (60%) out of the 15 isolates obtained from PSR2A-C/T/N plates were affiliated with the phylum *Actinobacteria* and only the remaining four isolates (27%) were assigned to the phylum *Bacteroidetes*. The proportion of the isolates assigned to the genus *Chryseobacterium* was higher in PSR2A-C/T plates (30%) than in PSR2A-C/T/N plates (7%). Isolates assigned to the genus *Flavobacterium* may only be recovered from PSR2A-C/T plates.

Bacteria were isolated from onion rhizosphere soil, which is also a rich source of *Flavobacterium* and *Chryseobacterium* (Nishioka, T., *et al.* 2014. Abstracts for Phytopathological Society of Japan Annual Meeting. p. 103, Hokkaido, in Japanese), on the above two media. The results obtained showed that 23 and 19 bacterial colonies with a yellow/orange color appeared on triplicate plates of PSR2A-C/T and PSR2A-C/T/N media, respectively (Table 2). Eighteen (78%) and 11 (58%) out of the yellow/orange-colored isolates obtained from the PSR2A-C/T and PSR2A-C/T/N plates, respectively, were affiliated with the *Bacteroidetes* phylum. The proportion of *Chryseobacterium* strains isolated from onion rhizosphere soil was higher in PSR2A-C/T plates (22%) than in PSR2A-C/T/N plates (16%), and *Flavobacterium* strains were only isolated from PSR2A-C/T plates. The reason why *Flavobacterium* strains did not appear on PSR2A-C/T/N medium was that rhizosphere-associated *Flavobacterium* may be sensitive to high concentrations of NaCl. Further studies on the physiology of *Flavobacterium* strains are needed in order to clarify this, and information on optimum NaCl concentrations for the growth of these strains is valuable for the selective isolation of *Flavobacterium* and *Chryseobacterium* strains from rhizosphere environments. In short, PSR2A-C/T is suitable as a medium for the isolation of *Flavobacterium* and *Chryseobacterium* from the rhizosphere. However, the isolation frequencies of the strains assigned to genus *Niabella* were relatively high from Welsh onion and onion rhizosphere soil. Further studies to reduce or prevent the growth of *Niabella* strains will be needed in order to improve the recovery rates of *Flavobacterium* and *Chryseobacterium* strains.

### Identification of strains recovered using optimized medium

**Fig. 1:**
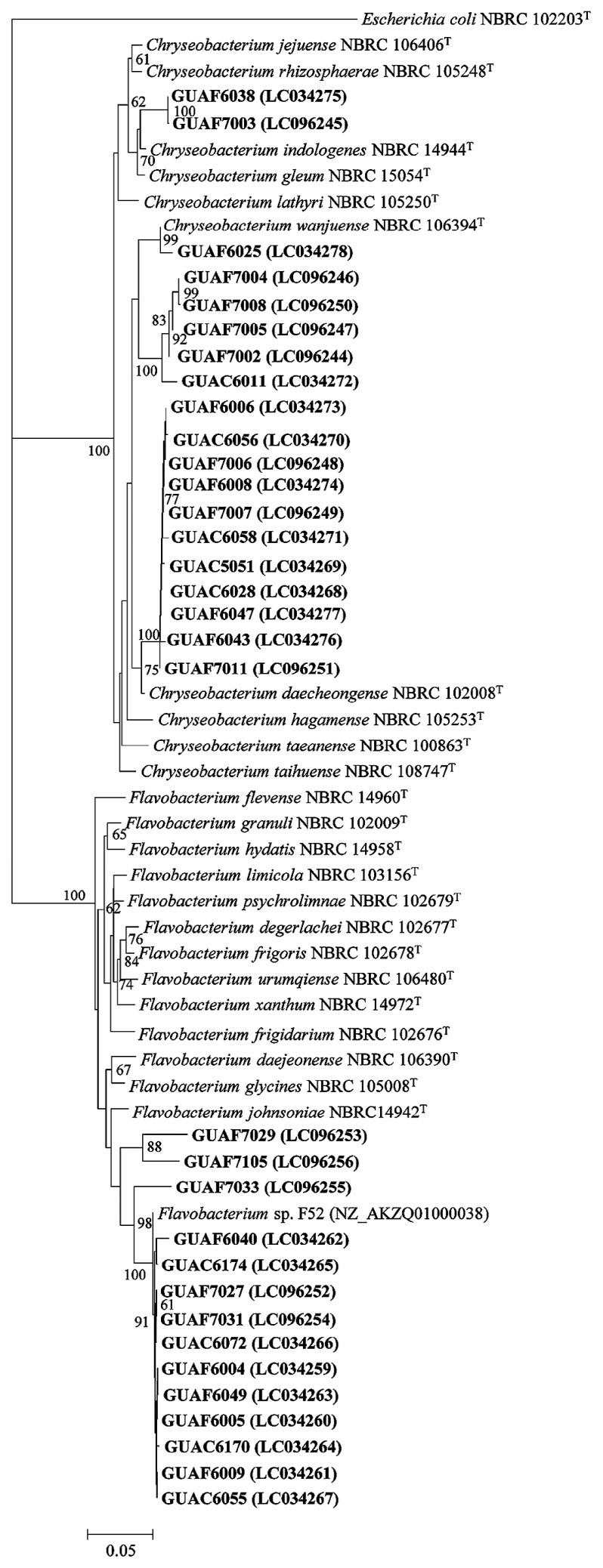
Phylogenetic relationships of partial 16S rDNA gene sequences obtained from strains related to *Flavobacterium* or *Chryseobacterium* isolated from Welsh onion and onion rhizosphere soil on phosphate separately autoclaved R2A supplemented with cycloheximide and tobramycin in isolation trials 3 and 4. The dendrogram was generated by the neighbor-joining method. The numbers on the branches represent the confidence intervals generated by bootstrapping with 1,000 replications; only values >60% are shown. The scale bar represents 0.1 substitutions per nucleotide position. Accession numbers are given in parentheses. *Escherichia coli* NBRC 102203^T^ was used as an outgroup.

All *Flavobacterium* strains were clustered with the species originating in soil (*F. johnsoniae* NBRC 14942T, *F. glycines* NBRC 105008T, and *F. daejeonense* NBRC 106390T) (Fig. 1). Kolton *et al.* (18, 19) previously reported that the genome sizes of soil/rhizosphere-associated *Flavobacterium* strains (4.0–6.1 Mbp) were nearly twice the size of aquatic *Flavobacterium* strains (2.86–3.96 Mbp), implying that terrestrial *Flavobacterium* strains possess higher metabolic capacities (*e.g.*, production of IAA, ACC deaminase, and antimicrobial compounds) than those of aquatic *Flavobacterium* strains. A comparative genomic analysis of *Flavobacterium* including the strains obtained in the present study is an important subject awaiting further investigation. Most *Chryseobacterium* strains were closely related to *C. daecheongense* NBRC 102008T and *C. wanjuense* NBRC 106394T (Fig. 1). Depolymerizing enzyme-producing *Chryseobacterium* strains have recently been discovered; for example, keratinase (8), mannanase (33), protease (48), and α-amylase (49). Biochemical analyses of enzymes produced by *Chryseobacterium* strains are required in order to ensure the usefulness of PSR2A-C/T medium for exploring beneficial microbial resources.

## Conclusion

**Fig. 2:**
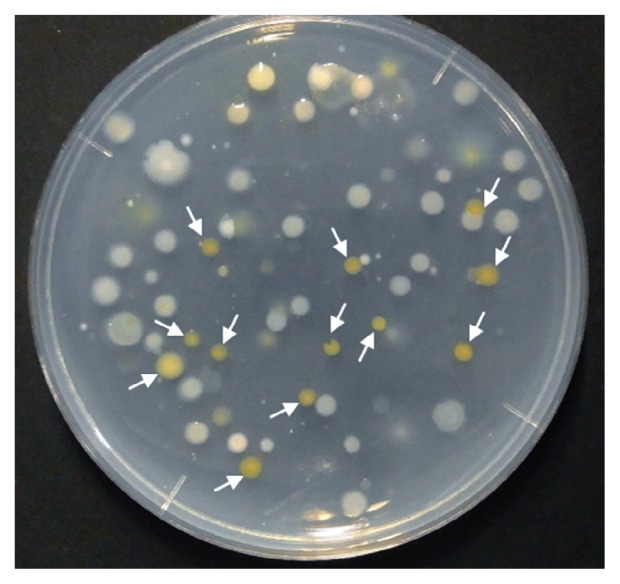
Bacterial colonies on phosphate separately autoclaved R2A supplemented with cycloheximide and tobramycin. White arrows indicate the example of bacterial colonies actually selected in this study.

We herein succeeded in developing optimized medium for the isolation of *Flavobacterium* and *Chryseobacterium* strains from rhizosphere soil. The following conditions are necessary: 1) the use of R2A agar as the basal medium, 2) supplementation with tobramycin and cycloheximide in order to prevent the growth of *Proteobacteria* strains and fungi, 3) autoclave phosphate separately to reduce inhibitory substances, 4) an incubation at 25°C for 14 d, and 5) the selection of yellow/orange-pigmented colonies (Fig. 2). This study may open new opportunities for further discovery of the beneficial properties of *Flavobacterium* and *Chryseobacterium* strains.

## Supplementary Material



## Figures and Tables

**Table 1:** Number of bacterial colonies on each medium in isolation trial 1.

Medium	Culture conditions		Number of colonies	
	
	Temperature	Periods	Yellow/orange	Total
R2A^a^	25°C	2 weeks	38	97
1/4-strength TSA^b^	25°C	2 weeks	1	59
1/4-strength TSA	30°C	3 d	33	49
1/10-strength TSA	25°C	2 weeks	2	109
1/10-strength TSA	30°C	3 d	2	182
SAA^c^	35°C	3 d	0	66
SAA-Gr^d^	35°C	3 d	0	43
SEA^e^	25°C	2 weeks	0	69

a Reasoner’s 2A agar

b Tryptic soy agar

c Schaedler anaerobe agar

d Schaedler anaerobe agar supplemented with gramicidin

e Soil-extract agar

**Table 2:** Bacterial isolates obtained from rhizosphere soil of Welsh onion and onion in isolation trial 1–4

Phylum	Genus	Number of species on medium									

		From Welsh onion rhizosphere								From onion rhizosphere	
	
		Trial 1		Trial 2		Trial 3		Trial 4			
			
		R2A^a^	1/4 TSA^b^	R2A	R2A-C/T^c^	R2A-C/T	PSR2A-C/T^d^	PSR2A-C/T	PSR2A-C/T/N^e^	PSR2A-C/T	PSR2A-C/T/N
*Bacteroidetes*	*Flavobacterium*						5	5		4	
	*Chryseobacterium*	1		1		4	8	6	1	5	3
	*Niabella*	2		2	14	9	2	4		6	6
	*Chitinophaga*							1	2	1	2
	*Niastella*	1				2					
	*Dyadobacter*								1		
	*Taibaiella*			1		1	1			2	
	*Flavitalea*					1					
*Proteobacteria*	*Sphingomonas*	22	1	6							1
	*Pseudoxanthomonas*		10							1	
	*Ralstonia*							3		2	
	*Sphingopyxis*	1		1					1		
	*Frateuria*	1									
	*Luteibacter*	1									
	*Lysobacter*	1									
	*Rhodanobacter*	1									
	*Pseudomonas*		1								
	*Sphingobium*		1								
	*Stenotrophomonas*			1			2				
	*Variovorax*								1		
*Actinobacteria*	*Microbacterium*	3	20				1		5		2
	*Leifsonia*			1					3	2	5
	*Mycobacterium*	1		1							
	*Aeromicrobium*	1									
	*Cellulosimicrobium*	1									
	*Rhodococcus*	1									
	*Terrabacter*							1			
	*Agrococcus*								1		
Total yellow/orange bacterial colonies		38	33	14	14	17	19	20	15	23	19
Total bacterial colonies		97	49	121	106	169	183	248	201	132	155

a Reasoner’s 2A agar

b 1/4-strength tryptic soy agar

c Reasoner’s 2A supplemented with cycloheximide and tobramycin

d Phosphate separately autoclaved R2A supplemented with cycloheximide and tobramycin

e Phosphate separately autoclaved R2A supplemented with cycloheximide, tobramycin, and NaCl
