# Supplementary material for: Development of Culture Medium for the Isolation of Flavobacterium and Chryseobacterium from Rhizosphere Soil
Source: Microbes Environ. 2016 Apr 20;31(2):104–10. doi: 10.1264/jsme2.ME15144 (PMC4912144; doi:10.1264/jsme2.ME15144)
Supplement: Supplementary file 1 [file 31_104_s1.pdf]

Supplemental Table 1 Closest relatives of partial 16S rRNA genes of the strains isolated in the isolation trial 1-4.

Strain	Closest relatives		Similarity (%)	Alignment	Isolation source
	Microoganism	Accession number			
**Isolation trial 1 (1/4 TSA[a])**					
GUAF2002	*Microbacterium* sp. 408	GU814036	100	740/740	Welsh onion rhizosphere soil
GUAF2003	*Microbacterium hydrocarbonoxydans*	EU373354	99	584/585	Welsh onion rhizosphere soil
GUAF2004	*Microbacterium hydrocarbonoxydans*	EU373354	99	788/791	Welsh onion rhizosphere soil
GUAF2005	*Microbacterium paraoxydans*	EU221351	99	809/810	Welsh onion rhizosphere soil
GUAF2006	*Microbacterium arabinogalactanolyticum*	KM210240	100	773/773	Welsh onion rhizosphere soil
GUAF2007	*Microbacterium hydrocarbonoxydans*	EU373354	99	699/704	Welsh onion rhizosphere soil
GUAF2008	*Microbacterium hydrocarbonoxydans*	EU373354	99	786/787	Welsh onion rhizosphere soil
GUAF2010	*Pseudomonas fluorescens*	KP253039	100	807/807	Welsh onion rhizosphere soil
GUAF2012	*Pseudoxanthomonas* sp. LHR-07	HE716933	100	785/785	Welsh onion rhizosphere soil
GUAF2013	*Microbacterium hydrocarbonoxydans*	EU373354	99	786/788	Welsh onion rhizosphere soil
GUAF2014	*Sphingobium japonicum* UT26S	AP010804	98	749/757	Welsh onion rhizosphere soil
GUAF2015	*Microbacterium paludicola*	AJ853909	98	724/735	Welsh onion rhizosphere soil
GUAF2019	*Microbacterium* sp. MA1	FJ357539	99	791/792	Welsh onion rhizosphere soil
GUAF2020	*Pseudoxanthomonas mexicana*	JX860406	99	800/802	Welsh onion rhizosphere soil
GUAF2022	*Microbacterium* sp. RU-24	KC405581	99	792/799	Welsh onion rhizosphere soil
GUAF2024	*Pseudoxanthomonas mexicana*	JX860406	100	804/804	Welsh onion rhizosphere soil
GUAF2025	*Microbacterium hydrocarbonoxydans*	EU373354	99	804/808	Welsh onion rhizosphere soil
GUAF2027	*Pseudoxanthomonas mexicana*	JX860406	100	840/840	Welsh onion rhizosphere soil
GUAF2028	*Microbacterium hydrocarbonoxydans*	EU373354	99	798/803	Welsh onion rhizosphere soil
GUAF2030	*Microbacterium hydrocarbonoxydans*	EU373354	99	845/846	Welsh onion rhizosphere soil
GUAF2031	*Pseudoxanthomonas* sp. LHR-07	HE716933	100	855/855	Welsh onion rhizosphere soil
GUAF2032	*Microbacterium hydrocarbonoxydans*	EU373354	99	788/791	Welsh onion rhizosphere soil
GUAF2033	*Pseudoxanthomonas mexicana*	JX860406	100	826/826	Welsh onion rhizosphere soil
GUAF2035	*Microbacterium hydrocarbonoxydans*	EU373354	99	841/842	Welsh onion rhizosphere soil
GUAF2036	*Microbacterium hydrocarbonoxydans*	EU714368	98	837/846	Welsh onion rhizosphere soil
GUAF2037	*Sphingomonas* sp. EM0332	EU448286	100	858/858	Welsh onion rhizosphere soil
GUAF2038	*Pseudoxanthomonas* sp. LHR-07	HE716933	99	854/855	Welsh onion rhizosphere soil
GUAF2039	*Microbacterium foliorum*	KP208636	99	842/846	Welsh onion rhizosphere soil
GUAF2040	*Pseudoxanthomonas mexicana*	JX860406	99	806/807	Welsh onion rhizosphere soil

GUAF2041	*Pseudoxanthomonas mexicana*	KM210271	99	831/832	Welsh onion rhizosphere soil
GUAF2042	*Pseudoxanthomonas* sp. LHR-07	HE716933	100	803/803	Welsh onion rhizosphere soil
GUAF2043	*Microbacterium xylanilyticum*	AJ853908	100	847/847	Welsh onion rhizosphere soil
GUAF2044	*Microbacterium hydrocarbonoxydans*	EU373354	99	827/828	Welsh onion rhizosphere soil
**Isolation trial 1 (R2A[b])**					
GUAF3001	*Niabella yanshanensis*	KC854959	99	797/800	Welsh onion rhizosphere soil
GUAF3002	*Chryseobacterium* sp. MN13.3d	AM159535	99	807/810	Welsh onion rhizosphere soil
GUAF3003	*Rhodococcus* sp. Eu-32	DQ386111	99	842/843	Welsh onion rhizosphere soil
GUAF3004	*Sphingomonas* sp. M37-VN10-2W	AB299579	100	833/833	Welsh onion rhizosphere soil
GUAF3005	*Lysobacter* sp. JC174	HF536576	99	848/854	Welsh onion rhizosphere soil
GUAF3006	*Mycobacterium neoaurum* VKM Ac-1815	CP006936	100	824/824	Welsh onion rhizosphere soil
GUAF3007	*Cellulosimicrobium cellulans*	X79453	100	837/837	Welsh onion rhizosphere soil
GUAF3009	*Sphingopyxis* sp. MC1	JN940802	100	844/844	Welsh onion rhizosphere soil
GUAF3010	*Sphingomonas* sp. EM0332	EU448286	100	834/834	Welsh onion rhizosphere soil
GUAF3012	*Sphingomonas* sp. EM0332	EU448286	100	836/836	Welsh onion rhizosphere soil
GUAF3013	*Sphingomonas* sp. M37-VN10-2W	AB299579	99	836/837	Welsh onion rhizosphere soil
GUAF3014	*Sphingomonas* sp. W2.10-2	JX458462	99	862/864	Welsh onion rhizosphere soil
GUAF3015	*Sphingomonas* sp. W2.10-2	JX458462	99	859/861	Welsh onion rhizosphere soil
GUAF3016	*Sphingomonas* sp. W2.10-2	JX458462	99	842/843	Welsh onion rhizosphere soil
GUAF3017	*Sphingomonas* sp. W2.10-2	JX458462	100	821/821	Welsh onion rhizosphere soil
GUAF3018	*Sphingomonas* sp. W2.10-2	JX458462	100	841/841	Welsh onion rhizosphere soil
GUAF3019	*Sphingomonas* sp. M37-VN10-2W	AB299579	99	836/837	Welsh onion rhizosphere soil
GUAF3020	*Sphingomonas* sp. W2.10-2	JX458462	100	821/821	Welsh onion rhizosphere soil
GUAF3021	*Sphingomonas* sp. W2.10-2	JX458462	100	821/821	Welsh onion rhizosphere soil
GUAF3022	*Luteibacter* sp. UR 6-04	KM253215	99	833/840	Welsh onion rhizosphere soil
GUAF3023	*Sphingomonas* sp. W2.10-2	JX458462	100	842/842	Welsh onion rhizosphere soil
GUAF3024	*Niabella yanshanensis*	KC854959	100	844/844	Welsh onion rhizosphere soil
GUAF3025	*Sphingomonas* sp. W2.10-2	JX458462	100	843/843	Welsh onion rhizosphere soil
GUAF3026	*Microbacterium* sp. HT-Z89-B1	KJ526938	100	834/834	Welsh onion rhizosphere soil
GUAF3027	*Aeromicrobium tamlense*	DQ411541	99	811/812	Welsh onion rhizosphere soil
GUAF3028	*Sphingopyxis* sp. TBD181	LC005606	100	834/834	Welsh onion rhizosphere soil
GUAF3029	*Sphingomonas* sp. M37-VN10-2W	AB299579	100	817/817	Welsh onion rhizosphere soil

GUAF3030	*Rhodanobacter lindaniclasticus*	AB245365	99	849/850	Welsh onion rhizosphere soil
GUAF3031	*Sphingomonas* sp. M37-VN10-2W	AB299579	100	845/845	Welsh onion rhizosphere soil
GUAF3032	*Sphingomonas* sp. W2.10-2	JX458462	100	843/843	Welsh onion rhizosphere soil
GUAF3034	*Sphingomonas* sp. M37-VN10-2W	AB299579	99	835/836	Welsh onion rhizosphere soil
GUAF3035	*Sphingopyxis* sp. TBD181	LC005606	100	834/834	Welsh onion rhizosphere soil
GUAF3036	*Caulobacter* sp. HWE-A01	JF722654	99	834/835	Welsh onion rhizosphere soil
GUAF3037	*Sphingomonas* sp. W2.10-2	JX458462	100	821/821	Welsh onion rhizosphere soil
GUAF3038	*Niastella* sp. KP03	AB329629	98	857/870	Welsh onion rhizosphere soil
GUAF3039	*Sphingomonas* sp. W2.10-2	JX458462	100	837/837	Welsh onion rhizosphere soil
GUAF3040	*Sphingomonas* sp. M37-VN10-2W	AB299579	100	813/813	Welsh onion rhizosphere soil
GUAF3041	*Microbacterium* sp. MA1	FJ357539	99	820/821	Welsh onion rhizosphere soil
GUAF3042	*Microbacterium oxydans*	DQ350825	100	851/851	Welsh onion rhizosphere soil
GUAF3043	*Frateuria terrea*	KC854841	99	829/837	Welsh onion rhizosphere soil
GUAF3044	*Sphingomonas* sp. W2.10-2	JX458462	100	810/810	Welsh onion rhizosphere soil
**Isolation trial 2 (R2A)**					
GUAF3153	*Niabella* sp. PD7-3	AB506121	100	848/848	Welsh onion rhizosphere soil
GUAF3154	*Sphingopyxis* sp. TBD181	LC005606	100	648/648	Welsh onion rhizosphere soil
GUAF3155	*Sphingomonas* sp. W2.10-2	JX458462	99	851/852	Welsh onion rhizosphere soil
GUAF3156	*Sphingomonas* sp. W2.10-2	JX458462	100	664/664	Welsh onion rhizosphere soil
GUAF3157	*Sphingomonas* sp. JB13	JF745870	99	799/801	Welsh onion rhizosphere soil
GUAF3159	*Sphingomonas* sp. W2.10-2	JX458462	100	844/844	Welsh onion rhizosphere soil
GUAF3160	*Taibaiella* sp. T1-10	KR078254	93	759/809	Welsh onion rhizosphere soil
GUAF3161	*Niabella yanshanensis*	KC854959	99	645/646	Welsh onion rhizosphere soil
GUAF3162	*Mycobacterium neoaurum*	FJ172311	99	586/587	Welsh onion rhizosphere soil
GUAF3163	*Stenotrophomonas* sp. ZWS18	KM051417	100	868/868	Welsh onion rhizosphere soil
GUAF3165	*Chryseobacterium* sp. 91A-561T	HG738132	97	812/830	Welsh onion rhizosphere soil
GUAF3166	*Leifsonia* sp. SaZR8	JQ806448	99	850/852	Welsh onion rhizosphere soil
GUAF3167	*Sphingomonas* sp. 8b-1	AY561541	99	753/755	Welsh onion rhizosphere soil
GUAF3169	*Sphingomonas* sp. 8b-1	AY561541	100	854/854	Welsh onion rhizosphere soil
**Isolation trial 2 (R2A-C/T[c])**					
GUAF4001	*Niabella yanshanensis*	KC854959	100	845/845	Welsh onion rhizosphere soil

GUAF4002	*Niabella yanshanensis*	KC854959	99	831/833	Welsh onion rhizosphere soil
GUAF4004	*Niabella* sp. PD7-3	AB506121	100	773/774	Welsh onion rhizosphere soil
GUAF4005	*Niabella* sp. PD7-3	AB506121	99	818/819	Welsh onion rhizosphere soil
GUAF4006	*Niabella yanshanensis*	KC854959	100	754/754	Welsh onion rhizosphere soil
GUAF4007	*Niabella yanshanensis*	KC854959	100	845/845	Welsh onion rhizosphere soil
GUAF4012	*Niabella yanshanensis*	KC854959	100	844/844	Welsh onion rhizosphere soil
GUAF4013	*Niabella yanshanensis*	KC854959	100	844/844	Welsh onion rhizosphere soil
GUAF4014	*Niabella* sp. PD7-3	AB506121	98	847/861	Welsh onion rhizosphere soil
GUAF4015	*Niabella yanshanensis*	KF307654	100	791/791	Welsh onion rhizosphere soil
GUAF4016	*Niabella yanshanensis*	KC854959	100	844/844	Welsh onion rhizosphere soil
GUAF4017	*Niabella yanshanensis*	KC854959	100	844/844	Welsh onion rhizosphere soil
GUAF4018	*Niabella* sp. PD7-3	AB506121	100	853/853	Welsh onion rhizosphere soil
GUAF4019	*Niabella yanshanensis*	KC854959	100	844/844	Welsh onion rhizosphere soil
**Isolation trial 3 (R2A-C/T)**					
GUAF7048	*Niabella yanshanensis*	KC854959	98	717/725	Welsh onion rhizosphere soil
GUAF7049	*Chryseobacterium* sp. 91A-612	KP271932	98	752/765	Welsh onion rhizosphere soil
GUAF7050	*Chryseobacterium* sp. 91A-593T	HG738134	98	676/686	Welsh onion rhizosphere soil
GUAF7051	*Chryseobacterium* sp. 91A-593T	HG738134	98	791/801	Welsh onion rhizosphere soil
GUAF7053	*Taibaiella koreensis*	KC252613	95	801/842	Welsh onion rhizosphere soil
GUAF7055	*Chryseobacterium daecheongense*	AJ457206	98	798/811	Welsh onion rhizosphere soil
GUAF7095	*Niabella yanshanensis*	KC854959	98	706/716	Welsh onion rhizosphere soil
GUAF7096	*Niabella yanshanensis*	KC854959	100	836/836	Welsh onion rhizosphere soil
GUAF7108	*Niabella* sp. PD7-3	AB506121	99	798/799	Welsh onion rhizosphere soil
GUAF7110	*Niabella yanshanensis*	KC854959	98	716/725	Welsh onion rhizosphere soil
GUAF7113	*Niabella yanshanensis*	KC854959	99	688/693	Welsh onion rhizosphere soil
GUAF7114	*Flavitalea* sp. KIS20-3	KM007101	94	650/688	Welsh onion rhizosphere soil
GUAF7115	*Niastella* sp. KP03	AB329629	99	675/676	Welsh onion rhizosphere soil
GUAF7117	*Niabella yanshanensis*	KC854959	98	676/684	Welsh onion rhizosphere soil
GUAF7119	*Niabella yanshanensis*	KC854959	98	804/816	Welsh onion rhizosphere soil
GUAF7120	*Niabella yanshanensis*	KF307654	100	716/716	Welsh onion rhizosphere soil
GUAF7121	*Niastella* sp. KP03	AB329629	99	754/756	Welsh onion rhizosphere soil

**Isolation trial 3 (PSR2A-C/T[d])**					
GUAF7001	*Stenotrophomonas maltophilia*	KF263488	99	760/761	Welsh onion rhizosphere soil
GUAF7002 (LC096244)	*Chryseobacterium* sp. 91A-593T	HG738134	98	796/805	Welsh onion rhizosphere soil
GUAF7003 (LC096245)	*Chryseobacterium* sp. JIP 108/83	AY468458	99	831/832	Welsh onion rhizosphere soil
GUAF7004 (LC096246)	*Chryseobacterium* sp. 91A-593T	HG738134	98	658/693	Welsh onion rhizosphere soil
GUAF7005 (LC096247)	*Chryseobacterium* sp. 91A-593T	HG738134	98	796/805	Welsh onion rhizosphere soil
GUAF7006 (LC096248)	*Chryseobacterium daecheongense*	AJ457206	98	736/748	Welsh onion rhizosphere soil
GUAF7007 (LC096249)	*Chryseobacterium daecheongense*	AJ457206	98	798/812	Welsh onion rhizosphere soil
GUAF7008 (LC096250)	*Chryseobacterium* sp. 91A-593T	HG738134	98	606/616	Welsh onion rhizosphere soil
GUAF7011 (LC096251)	*Chryseobacterium daecheongense*	AJ457206	98	776/789	Welsh onion rhizosphere soil
GUAF7018	*Stenotrophomonas maltophilia*	KF263488	99	811/812	Welsh onion rhizosphere soil
GUAF7027 (LC096252)	*Flavobacterium* sp. THWCSN41	AM888190	99	842/843	Welsh onion rhizosphere soil
GUAF7029 (LC096253)	*Flavobacterium* sp. C-2	HQ188912	99	795/801	Welsh onion rhizosphere soil
GUAF7031 (LC096254)	*Flavobacterium* sp. THWCSN41	AM888190	99	828/829	Welsh onion rhizosphere soil
GUAF7033 (LC096255)	*Flavobacterium* sp. WG1	FN547415	98	731/743	Welsh onion rhizosphere soil
GUAF7073	*Microbacterium testaceum*	AF474330	99	817/818	Welsh onion rhizosphere soil
GUAF7105 (LC096256)	*Flavobacterium* sp. CC-JY-6	DQ239767	97	778/798	Welsh onion rhizosphere soil
GUAF7120	*Niabella yanshanensis*	KF307654	99	689/690	Welsh onion rhizosphere soil
GUAF7122	*Taibaiella koreensis*	KC252613	94	647/683	Welsh onion rhizosphere soil
**Isolation trial 4 (PSR2A-C/T)**					
GUAF6003	*Niabella yanshanensis*	KC854959	99	843/844	Welsh onion rhizosphere soil
GUAF6004 (LC034259)	*Flavobacterium* sp. THWCSN41	AM888190	99	841/842	Welsh onion rhizosphere soil
GUAF6005 (LC034260)	*Flavobacterium* sp. THWCSN41	AM888190	99	854/856	Welsh onion rhizosphere soil
GUAF6006 (LC034273)	*Chryseobacterium daecheongense*	AJ457206	98	817/832	Welsh onion rhizosphere soil
GUAF6007	*Niabella yanshanensis*	KC854959	100	830/830	Welsh onion rhizosphere soil
GUAF6008 (LC034274)	*Chryseobacterium daecheongense*	AJ457206	98	803/815	Welsh onion rhizosphere soil
GUAF6009 (LC034261)	*Flavobacterium* sp. THWCSN41	AM888190	99	860/861	Welsh onion rhizosphere soil
GUAF6018	*Ralstonia mannitolilytica*	KF150335	95	705/737	Welsh onion rhizosphere soil
GUAF6020	*Ralstonia mannitolilytica*	KF150335	96	628/650	Welsh onion rhizosphere soil
GUAF6022	*Terrabacter* sp. MUSC78T	KF682157	98	838/852	Welsh onion rhizosphere soil
GUAF6023	*Chitinophaga filiformis*	JX280517	98	809/821	Welsh onion rhizosphere soil
GUAF6025 (LC034278)	*Chryseobacterium wanjuense*	DQ256729	99	802/803	Welsh onion rhizosphere soil

GUAF6037	*Niabella* sp. PD7-3	AB506121	100	866/866	Welsh onion rhizosphere soil
GUAF6038 (LC034275)	*Chryseobacterium* sp. JIP 108/83	AY468458	100	854/854	Welsh onion rhizosphere soil
GUAF6039	*Niabella yanshanensis*	KC854959	100	843/843	Welsh onion rhizosphere soil
GUAF6040 (LC034262)	*Flavobacterium johnsoniae*	EU221404	98	737/745	Welsh onion rhizosphere soil
GUAF6043 (LC034276)	*Chryseobacterium daecheongense*	AJ457206	97	843/862	Welsh onion rhizosphere soil
GUAF6047 (LC034277)	*Chryseobacterium daecheongense*	AJ457206	98	867/881	Welsh onion rhizosphere soil
GUAF6049 (LC034263)	*Flavobacterium* sp. THWCSN41	AM888190	99	877/879	Welsh onion rhizosphere soil
GUAF6074	*Ralstonia pickettii*	CP001645	99	816/817	Welsh onion rhizosphere soil
GUAC6011 (LC034272)	*Chryseobacterium* sp. 91A-561T	HG738132	99	812/819	Onion rhizosphere soil
GUAC6025	*Niabella* sp. PD7-3	AB506121	97	834/858	Onion rhizosphere soil
GUAC6026	*Taibaiella koreensis*	KC252613	95	761/800	Onion rhizosphere soil
GUAC6027	*Taibaiella koreensis*	KC252613	94	800/850	Onion rhizosphere soil
GUAC6028 (LC034268)	*Chryseobacterium daecheongense*	AJ457206	97	793/810	Onion rhizosphere soil
GUAC6034	*Leifsonia* sp. SIU	KJ191763	99	671/673	Onion rhizosphere soil
GUAC6036	*Pseudoxanthomonas* sp. CHNTR38	DQ337597	95	815/852	Onion rhizosphere soil
GUAC6038	*Leifsonia* sp. SIU	KJ191763	99	829/834	Onion rhizosphere soil
GUAC6047	*Ralstonia pickettii* 12D	CP001645	100	852/852	Onion rhizosphere soil
GUAC6048	*Ralstonia pickettii* 12D	CP001645	100	838/838	Onion rhizosphere soil
GUAC6051 (LC034269)	*Chryseobacterium daecheongense*	AJ457206	98	791/807	Onion rhizosphere soil
GUAC6052	*Niabella yanshanensis*	KC854959	98	830/841	Onion rhizosphere soil
GUAC6053	*Niabella* sp. PD7-3	AB506121	100	866/866	Onion rhizosphere soil
GUAC6054	*Niabella yanshanensis*	KC854959	99	842/843	Onion rhizosphere soil
GUAC6055 (LC034267)	*Flavobacterium* sp. THWCSN41	AM888190	99	860/862	Onion rhizosphere soil
GUAC6056 (LC034270)	*Chryseobacterium daecheongense*	AJ457206	98	818/833	Onion rhizosphere soil
GUAC6058 (LC034271)	*Chryseobacterium daecheongense*	AJ457206	98	856/870	Onion rhizosphere soil
GUAC6072 (LC034266)	*Flavobacterium johnsoniae*	EU221404	99	779/780	Onion rhizosphere soil
GUAC6109	*Niabella yanshanensis*	KC854959	98	820/829	Onion rhizosphere soil
GUAC6113	*Niabella yanshanensis*	KC854959	98	789/797	Onion rhizosphere soil
GUAC6114	*Chitinophaga* sp. BS20	JF806524	94	987/728	Onion rhizosphere soil
GUAC6170 (LC034264)	*Flavobacterium* sp. THWCSN41	AM888190	99	864/867	Onion rhizosphere soil
GUAC6174 (LC034265)	*Flavobacterium* sp. THWCSN41	AM888190	99	799/801	Onion rhizosphere soil

Isolation trial 4(PSR2A-C/T/N[e])

GUAF5001	*Microbacterium* sp. W1.10-194	JX458442	99	840/841	Welsh onion rhizosphere soil
GUAF5002	*Microbacterium* sp. W1.10-194	JX458442	99	840/841	Welsh onion rhizosphere soil
GUAF5003	*Microbacterium oleivorans*	EF522131	100	689/698	Welsh onion rhizosphere soil
GUAF5024	*Chryseobacterium wanjuense*	DQ256729	99	845/850	Welsh onion rhizosphere soil
GUAF5027	*Variovorax soli* NBRC 106424	DQ432053	99	859/861	Welsh onion rhizosphere soil
GUAF5028	*Agrococcus* sp. SS14.8	KC160779	100	850/850	Welsh onion rhizosphere soil
GUAF5029	*Leifsonia xyli*	HQ530514	99	812/814	Welsh onion rhizosphere soil
GUAF5030	*Chitinophaga* sp. R3-710	JQ659659	98	837/849	Welsh onion rhizosphere soil
GUAF5031	*Chitinophaga* sp. LWH185	KC854879	99	843/850	Welsh onion rhizosphere soil
GUAF5032	*Microbacterium kyungheense*	JX997973	99	850/851	Welsh onion rhizosphere soil
GUAF5035	*Microbacterium oleivorans*	EF522131	99	821/823	Welsh onion rhizosphere soil
GUAF5085	*Leifsonia* sp. SaZR8	JQ806448	99	697/698	Welsh onion rhizosphere soil
GUAF5086	*Leifsonia* sp. SaZR8	JQ806448	99	846/850	Welsh onion rhizosphere soil
GUAF5087	*Dyadobacter fermentans* DSM 18053	CP001619	99	794/796	Welsh onion rhizosphere soil
GUAF5119	*Sphingopyxis* sp. TBD181	LC005606	100	814/814	Welsh onion rhizosphere soil
GUAC5008	*Niabella* sp. PD7-3	AB506121	99	846/851	Onion rhizosphere soil
GUAC5009	*Niabella* sp. PD7-3	AB506121	99	846/852	Onion rhizosphere soil
GUAC5010	*Niabella* sp. PD7-3	AB506121	98	852/863	Onion rhizosphere soil
GUAC5012	*Microbacterium paraoxydans*	EU221351	99	858/861	Onion rhizosphere soil
GUAC5013	*Chitinophaga arvensicola*	AB681050	99	805/811	Onion rhizosphere soil
GUAC5014	*Chitinophaga terrae* Kim and Jung 2007	KF150367	94	823/867	Onion rhizosphere soil
GUAC5015	*Niabella yanshanensis*	KC854959	98	826/835	Onion rhizosphere soil
GUAC5053	*Leifsonia* sp. SIU	KJ191763	99	833/840	Onion rhizosphere soil
GUAC5054	*Leifsonia* sp. SIU	KJ191763	99	860/862	Onion rhizosphere soil
GUAC5057	*Leifsonia xyli*	HQ530514	99	850/853	Onion rhizosphere soil
GUAC5058	*Chryseobacterium scophthalmum*	KC178594	98	835/852	Onion rhizosphere soil
GUAC5060	*Chryseobacterium* sp. 91A-561T	HG738132	98	842/854	Onion rhizosphere soil
GUAC5061	*Microbacterium hydrocarbonoxydans*	AJ698726	100	819/819	Onion rhizosphere soil
GUAC5062	*Sphingomonas* sp. M37-VN10-2W	AB299579	98	861/872	Onion rhizosphere soil
GUAC5063	*Leifsonia* sp. SIU	KJ191763	99	821/828	Onion rhizosphere soil
GUAC5064	*Leifsonia* sp. SIU	KJ191763	99	861/893	Onion rhizosphere soil
GUAC5099	*Chryseobacterium indologenes*	EU221399	99	799/804	Onion rhizosphere soil
GUAC5111	*Niabella* sp. PD7-3	AB506121	99	845/850	Onion rhizosphere soil
GUAC5112	*Niabella yanshanensis*	KC854959	98	763/771	Onion rhizosphere soil

[a] 1/4-strength tryptic soy agar

[b] Reasoner's 2A agar

[c] Reasoner's 2A supplemented with cycloheximide and tobramycin

[d] Phosphate separately autoclaved R2A supplemented with cycloheximide and tobramycin

[e] Phosphate separately autoclaved R2A supplemented with cycloheximide, tobramycin, and NaCl
